# Combining adverse pregnancy and perinatal outcomes for women exposed to antiepileptic drugs during pregnancy, using a latent trait model

**DOI:** 10.1186/s12884-016-1190-7

**Published:** 2017-01-06

**Authors:** Xuerong Wen, Abraham Hartzema, Joseph A. Delaney, Babette Brumback, Xuefeng Liu, Robert Egerman, Jeffrey Roth, Rich Segal, Kimford J. Meador

**Affiliations:** 1 Health Outcomes, College of Pharmacy, University of Rhode Island, 7 Greenhouse Rd., Kingston, RI 02881 USA; 2 Department of Pharmaceutical Outcome and Policy, University of Florida, Gainesville, FL USA; 3 Department of Epidemiology, University of Washington, Seattle, WA USA; 4 Department of Biostatistics, University of Florida, Gainesville, FL USA; 5 Department of Biostatistics & Epidemiology, Systems, Population and Leadership, University of Michigan, Ann Arbor, MI 48109 USA; 6 Department of Obstetrics & Gynecology, University of Florida, Gainesville, FL USA; 7 Department of Pediatrics, University of Florida, Gainesville, FL USA; 8 Department of Neurology & Neurological Sciences, Stanford University, Stanford, CA USA

**Keywords:** Latent trait model, Antiepileptic drugs, Valproate, Adverse pregnancy outcome, Adverse perinatal outcome, Combining outcomes

## Abstract

**Background:**

Application of latent variable models in medical research are becoming increasingly popular. A latent trait model is developed to combine rare birth defect outcomes in an index of infant morbidity.

**Methods:**

This study employed four statewide, retrospective 10-year data sources (1999 to 2009). The study cohort consisted of all female Florida Medicaid enrollees who delivered a live singleton infant during study period. Drug exposure was defined as any exposure to Antiepileptic drugs (AEDs) during pregnancy. Mothers with no AED exposure served as the AED unexposed group for comparison. Four adverse outcomes, birth defect (BD), abnormal condition of new born (ACNB), low birth weight (LBW), and pregnancy and obstetrical complication (PCOC), were examined and combined using a latent trait model to generate an overall severity index. Unidimentionality, local independence, internal homogeneity, and construct validity were evaluated for the combined outcome.

**Results:**

The study cohort consisted of 3183 mother-infant pairs in total AED group, 226 in the valproate only subgroup, and 43,956 in the AED unexposed group. Compared to AED unexposed group, the rate of BD was higher in both the total AED group (12.8% vs. 10.5%, *P* < .0001), and the valproate only subgroup (19.6% vs. 10.5%, *P* < .0001). The combined outcome was significantly correlated with the length of hospital stay during delivery in both the total AED group (Rho = 0.24, *P* < .0001) and the valproate only subgroup (Rho = 0.16, *P* = .01). The mean score for the combined outcome in the total AED group was significantly higher (2.04 ± 0.02 vs. 1.88 ± 0.01, *P* < .0001) than AED unexposed group, whereas the valproate only subgroup was not.

**Conclusions:**

Latent trait modeling can be an effective tool for combining adverse pregnancy and perinatal outcomes to assess prenatal exposure to AED, but evaluation of the selected components is essential to ensure the validity of the combined outcome.

**Electronic supplementary material:**

The online version of this article (doi:10.1186/s12884-016-1190-7) contains supplementary material, which is available to authorized users.

## Keypoints

- AEDs have significant effects on all four component birth outcomes, and as well as the combined outcome.
- Valproate has significant effects on two out of four component outcomes, and no association with the combined outcome.
- Latent Trait Modeling is an effective tool to combine rare birth defect outcomes.
- Evaluation of selected components is essential to ensure the validity of the combined outcome.

## Background

Birth defects (BDs), involving major congenital malformation (MCM) and minor anomaly (MA) are the leading causes of infant mortality, morbidity, and years of potential life lost. In the USA, the association of infant BDs and pregnancy and obstetrical complications (PCOCs) with maternal exposure to antiepileptic drugs (AEDs) has been investigated extensively [1–3]. However, the rare occurrence of BDs, abnormal condition of new born (ACNBs), and PCOCs limits the power of most published studies, and makes study results inconclusive [4–6]. A joint model for combining individual outcomes is proposed to improve the efficiency and power of BD studies [7].

Latent variable models have increasingly been applied in medical research, including measurement of quality of life, diagnostic testing, survival analysis, and joint modeling of longitudinal data [8]. Latent variables are unobserved variables that can only be assessed indirectly by observable manifest variables. A latent variable model is a statistical approach that uses a set of observable manifest variables to derive one or more unobsersable variables. In latent variable model with a latent trait setting, the manifest variables are discrete, including dichotomous, nominal, or ordinal variables, whereas, the latent variables are continuous variables and can be assumed as normally or log-normally distributed [9]. An important assumption for latent variable model is the “local independence”, defined as that the manifest variables are conditionally independent upon a given latent variable, and the relationship among the manifest variables is fully explained by the latent variable [10]. A latent variable model in a latent trait setting was developed for this study to combine individual BD outcomes and generate an infant morbidity index [11]. This model combines four infant morbidity outcomes and generates a continuous index representing the infant’s propensity for morbidity [11]. Application of this model to combine rare adverse pregnancy and perinatal outcomes in drug safety studies may increase statistical power and improve efficiency of studies investigating low prevalence sequelae.

A debate remains over the use of combined or individual outcomes in drug safety studies. A combined outcome may lead to incorrect results and threaten the validity of the study if the components are selected inappropriately [12, 13]. Therefore, the combined outcome must be evaluated in terms of conceptualization of the composite outcome [12], and appropriate properties of the latent variable, such as local independence, construct validity and reliability [14].

The objective of this study is to apply a latent trait model to generate a valid combined outcome (adverse perinatal and pregnancy outcome; APO) to assess the overall adverse pregnancy and perinatal risks for mothers and infants exposed to AEDs.

## Methods

### Data sources

This study used four statewide, retrospective 10-year databases: Florida Medicaid claims, Florida Birth Vital Statistics, Florida Birth Anomalies, and Florida Hospital Discharge Inpatient and Outpatient records (January 1, 1999–December 31, 2009).

### Study population

This study includes all female Florida Medicaid enrollees who delivered a live singleton infant between April 1, 2000 and December 31, 2009. Exclusion criteria for maternal-infant pairs are: mothers with dual eligibility for Medicare, HMO, or private insurance; mothers having multiple births (twins or higher order); mothers with diabetes mellitus (ICD-9 codes: 249.x, 250.x, 790.29, or use of any anti-diabetics during baseline), hypertension (ICD-9 codes: 401.x, 416.x, 796.2, 997.91, 459.3, or antihypertensive drug use during baseline), or HIV pre-pregnancy (ICD-9 codes: 042, 079.53, V08, V01.79, 795.71, or use of any antiretroviral therapy); infants who were twins, triplets, quadruplets or more; infants with birth weight lower than 350 g or higher than 6000 g; mothers or infants with critical information missing (e.g., birth weight, demographics, or medical information).

### Study design

The index date is the infant’s birth date. The drug exposure window was defined as the preceding 9-month pregnancy period after the first day of the last menstrual date. A six month baseline period before the first date of the last menstrual date was utilized to determine the baseline demographic and clinical characteristics. BD outcomes were detected 0–365 days after live birth.

### Exposure

Drug exposure was determined from Medicaid pharmacy claims using national drug codes. Two drug exposure groups, valproate and AEDs (including valproate), were employed to develop two scenarios with different patterns of association with the four component outcomes. Valproate use was defined as prescriptions dispensed for valproate, sodium valproate, or divalproex. AEDs included: carbamazepine, ethosuximide, felbamate, gabapentin, lamotrigine, levetiracetam, oxcarbazepine, phenobarbital, phenytoin, pregabalin, primidone, tiagabine, topiramate, valproate, and zonisamide.

The birth anomalies are related to exposure time during pregnancy: [15] MCM associates with teratogen exposure in the first trimester [16], and MA and LBW relate to the maternal drug exposure in the third trimester [15, 17]. Therefore, maternal drug exposure during the entire pregnancy can affect the combined outcome. The prenatal drug exposure window was established as the period of 14 days before the first day of the mother’s last menstrual period to the infant’s birth date. The drug exposure was defined as any one dose of the drugs listed above dispensed during the exposure window, including which drug was dispensed prior to the exposure window and its days of supply covers at least one day of the exposure window. Adding 14 days prior to the pregnancy takes into account the conception period and the residual effects of AEDs. Sensitivity analysis was conducted to examine the effects of different drug exposure windows on the combined outcome.

### Component outcomes

We investigated four adverse pregnancy and infant outcomes: BD (involving MCM and MA), abnormal condition of new born (ACNB), LBW, and PCOC from multiple data sources. The operational definition for each component outcome was listed in Additional file 1: Table S1. MCMs and MAs were collected for 365 days following birth using the 9^th^ edition of the International Classification of Diseases-Clinical Modification (ICD-9 CM) code (740–759.9) from Florida Hospital Discharge Inpatient and Outpatient data. It has been confirmed that Hospital Discharge data, along with other Children’s Medical Services diagnostic information, efficiently enhanced case ascertainment for BD cases from Florida Birth Vital Statistics data [18–20]. ACNB and birth weight were obtained from Florida Birth Vital Statistics. The common conditions of ACNBs include anemia, birth injury, fetal alcohol syndrome, hyaline membrane disease, and assisted ventilation. Birth weight was categorized into four levels: Extremely Low Birth Weight (ELBW, 350–999 g), Very Low Birth Weight (VLBW, 1000–1499 g), Low Birth Weight (LBW, 1500–2499 g) and Normal Birth Weight (NBW, 2500–5999 g). PCOCs were identified either from Florida Birth Vital Statistics data or using ICD-9-CM and Current Procedural Terminology codes from Medicaid inpatient and outpatient claims data depending upon the extent of the validity and reliability of these data sources as reported in previous studies [21–25]. Gestational hypertension, preeclampsia, and eclampsia were identified using ICD-9-CM codes from hospital discharge data [22, 23]. Preterm birth was operationally defined as gestational age less than 37 weeks [24]. Gestational age was computed from the infant birth date and mother’s last menstrual period. To identify obstetrical conditions, we defined cesarean delivery and forceps or vacuum extractor delivery from either birth certificates or ICD-9-CM codes in hospital discharge data, if it was missing in the birth certificates. Postpartum hemorrhage was identified solely using ICD-9-CM codes in hospital discharge data due to poor validity of birth certificate data on pregnancy complications and obstetric events [25].

Selected component outcomes were evaluated for similarity of importance, frequency rate, and treatment effect. The importance of the component outcome was assessed by computing Spearman correlations between individual outcomes and a clinically meaningful endpoint, defined as infant’s length of hospital stay following delivery [26].

### Reference group and covariates

A reference group, defined as infants with no maternal exposure to any AEDs during pregnancy and termed “AED unexposed group”, was selected for the estimation of treatment effects of the combined and component outcomes. The potential confounding factors were controlled using propensity score matching techniques. Previous studies have documented that common risk factors for adverse maternal and infant outcomes include socioeconomic status, infant gender, maternal age, race, BMI, smoking, alcohol consumption, parity, and drug exposure during pregnancy [27–30]. Significant teratogens such as alcohol and tobacco were controlled for during treatment effect assessment [31–36]. Other medical indications documented as teratogens in previous studies were also controlled in this study [37, 38]. Demographic characteristics were identified from birth certificates, whereas co-morbidities or co-medications during pregnancy were identified using ICD-9-CM and National Drug Codes from Hospital Discharge data.

### Combining outcomes using latent trait modeling

The statistical inference and mathematical algorithm for the model have been described elsewhere [39]. An important assumption of the model is “local independence”, defined as an independence of manifest outcomes conditioned on latent variables [11]. Estimated Generalized Nonlinear Least Squares estimation was employed to obtain the parameters involved in the latent trait model [11, 40]. The derivative process for the combined outcome is as follows:

- Step 1. Calculate initial estimates of the model parameters. First, we selected initial estimates to make the iteration process converge. We obtained 32 independent levels by combining 3 dichotomous component outcomes: BD (Yes/No), ACNB (Yes/No), PCOC (Yes/No), and 1 polytomous component outcome: Birth Weight (BW): 2500 ~ 5999 g, 1500 ~ 2499 g, 1000 ~ 1499 g, 350 ~ 999 g. The frequencies and proportions for each level of the combination of four component outcomes were calculated and utilized to deduce the initial estimates of the model parameters.
- Step 2. Derive the final estimates of the model parameters. Using the set of initial values and the modified Gauss-Newton algorithm, final estimates of the model parameters were obtained. The modified Gauss–Newton algorithm was run in SAS Proc IML, starting from the initialized value at iteration 0, until the difference of the last two estimates was less than 10^−9^. All final parameters were estimated from the iteration process.
- Step 3: Calculate the conditional probabilities given the latent variable S for each component outcome. Substituting the final estimates into the latent trait model, we calculated expected probabilities and counts for each level of the combination of four component outcomes.
- Step 4: Derive the combined outcome, the severity index of adverse perinatal and pregnancy outcome (APO). Substituting final estimates and conditional probabilities into the latent trait model, we further obtained the posterior distribution of latent variable S, and the mean of the posterior distribution (*ŝ*). The final estimate, APO, is a rescaled *ŝ*, to adapt for measurement of severity of health status.

### Evaluation of combined outcome

Local independence of four component outcomes was assessed using Yen’s Q statistics [41]. Validity and reliability of the combined outcome were evaluated using factor analysis and Spearman correlation [42, 43].

### Statistical analysis

Continuous variables were compared using a student *t* test, and categorical variables were examined using a chi-square test. Spearman correlation was calculated for discrete data, and Pearson correlation was calculated for continuous variables that are normally distributed. Multivariate logistic modeling was used to obtain propensity scores and assess the effects of drug use for each component outcome. Latent trait modeling was employed to combine four component outcomes into a severity index.

Statistical analysis was conducted using SAS 9.3 (Cary, NC). *P* < 0.05 was considered a statistically significant difference, except where *P* < 0.025 was deemed significant after Bonferroni correction for two comparisons.

## Results

After applying all inclusion and exclusion criteria, the final study cohort consisted of 3183 mother-infant pairs in the AED exposure group, 226 mother-infant pairs in the valproate exposure subgroup, and 43,956 mother-infant pairs in the AED unexposed group. A comparison of the demographic and clinical characteristics of the three groups is presented in Table 1, and the characteristics of all study populations, as well as missing data, were presented in Additional file 1: Table S2. The detailed data about AED exposure in pregnant women in Florida Medicaid has been published in elsewhere [44].

**Table 1:** Demographic and Clinical Characteristics of Study Participants. Obtained from Florida Birth Vital Statistics or Medicaid Claims Data

Characteristics	Valproate Sub-group *N* = 226	Total AED Group^a^ *N* = 3183	AED Unexposed Group *N* = 43,956	*P* Value**	*P* Value***
Maternal age at infant born, Mean ± SD	25.9 ± 6.4	26.5 ± 6.0	24.6 ± 5.2	<.0001	0.0014
Father’s age at infant birth, Mean ± SD	52.1 ± 33.2	47.5 ± 30.7	43.3 ± 29.7	<.0001	<.0001
Mother’s Race, *N* (%)					
White	177 (72)	2200 (69)	20,333 (46)	<.0001	<.0001
Black	27 (11)	429 (13)	13,991 (32)		
Others	41 (17)	552 (17)	9550 (22)		
Father’s Race, *N* (%)					
White	95 (39)	1455 (46)	15,669 (36)	<.0001	<.0001
Black	31 (13)	327 (10)	9733 (22)		
Others	32 (13)	474 (15)	8024 (18)		
Father’s education level, *N* (%)					
Above High School	66 (40)	765 (33)	14,219 (41)	<.0001	0.7107
Mother’s previous adverse pregnancy experience, *N* (%)	1 (0.7)	23 (1)	307 (2)	0.0237	0.3482
Mother’s receipt of any prenatal care, *N* (%)	143 (99)	2172 (99)	18,590 (98)	0.5571	0.0246
Mother’s total number of prenatal visits, Mean ± SD	11.1 ± 18.2	11.3 ± 16.9	8.8 ± 15.7	<.0001	0.0059
Mother’s marital status, Yes, *N* (%)	86 (35)	1137 (36)	18,041 (41)	<.0001	0.1679
Mother’s parity (previous live births), Mean ± SD	1.1 ± 1.3	1.3 ± 5.1	1.7 ± 3.7	<.0001	<.0001
Mother’s tobacco use, *N* (%)	74 (31)	936 (30)	7133 (16)	<.0001	<.0001
Mother’s average tobacco use, Mean ± SD	4.8 ± 15.9	4.0 ± 13.5	1.7 ± 9.0	<.0001	0.0028
Mother’s alcohol use, *N* (%)	3 (1)	38 (1)	175 (0.4)	<.0001	0.0183
Mother’s education level, *N* (%)					
Above High School	86 (37)	1173 (37)	16,772 (39)	0.0980	0.5377
Infant male gender, *N* (%)	120 (49)	1549 (49)	19,048 (43)	<.0001	0.0001
Mother’s previous gestational diabetes, *N* (%)	6 (4)	84 (4)	604 (3)	0.1714	0.4170
Mother’s previous gestational hypertension, *N* (%)	6 (3)	138 (5)	1240 (3)	<.0001	0.2559
Mother’s previous cesarean, *N* (%)	17 (13)	306 (14)	3056 (17)	0.0005	0.1819
Mother’s Epilepsy diagnosis during baseline and pregnancy, *N* (%)	60 (24)	571 (18)	81 (0.2)	<.0001	<.0001
Mother’s Anxiety diagnosis during baseline and pregnancy, *N* (%)	15 (6)	230 (7)	218 (0.5)	<.0001	<.0001
Mother’s Neural Pain diagnosis during baseline and pregnancy, *N* (%)	0 (0)	27 (0.9)	27 (0.1)	<.0001	>.999
Mother’s Bipolar diagnosis during baseline and pregnancy, *N* (%)	56 (23)	444 (14)	499 (1.1)	<.0001	<.0001
Mother’s Depression diagnosis during baseline and pregnancy, *N* (%)	21 (9)	328 (10.3)	743 (1.7)	<.0001	<.0001
Mother’s Migraine diagnosis during baseline and pregnancy, *N* (%)	12 (5)	96 (3)	173 (0.4)	<.0001	<.0001
Mother’s mental disorder diagnoses during baseline and pregnancy, *N* (%)	90 (37)	1118 (35)	3777 (9)	<.0001	<.0001
Mother’s antipsychotic exposure during pregnancy, *N* (%)	50 (20)	338 (11)	275 (0.6)	<.0001	<.0001
Mother’s antidepressants exposure during pregnancy, *N* (%)	90 (37)	886 (28)	1281 (3)	<.0001	<.0001
Mother’s folic acid use during pregnancy, *N* (%)	150 (61)	1965 (62)	17,948 (41)	<.0001	<.0001
Mother’s anxiolytics (including sedatives and hypnotics) exposure during pregnancy, *N* (%)	76 (31)	1832 (58)	1452 (3)	<.0001	<.0001
Number of hospitalization for seizure during pregnancy, Median (min, max)	0 (0, 4)	0 (0, 6)	0 (0, 3)	<.0001	<.0001
Number of physician visits with seizure diagnoses during pregnancy, Median (min, max), *N* (%)	0 (0, 3)	0 (0, 5)	0 (0, 6)	<.0001	0.0208
Mother’s infection and parasitic diagnosis during baseline and pregnancy, *N* (%)^b^	30 (12)	355 (11)	2959 (7)	<.0001	0.0006
Mother’s antibiotics exposure during pregnancy, *N* (%)	117 (48)	1418 (45)	13,854 (32)	<.0001	<.0001

^a^By definition, total AED group includes the patients who used valproate

^b^Include including: Virus, Rubella, Cytomegalovirus, HIV, Syphilis, Herpes simplex virus, Toxoplamosis, Varicella virus, Venezuelan equine encephalitis virus, Phenylketonuria, Hypoxia

**Compared between total AED group and AED unexposed group

***Compared between valproate subgroup and AED unexposed group

**Fig. 1:**
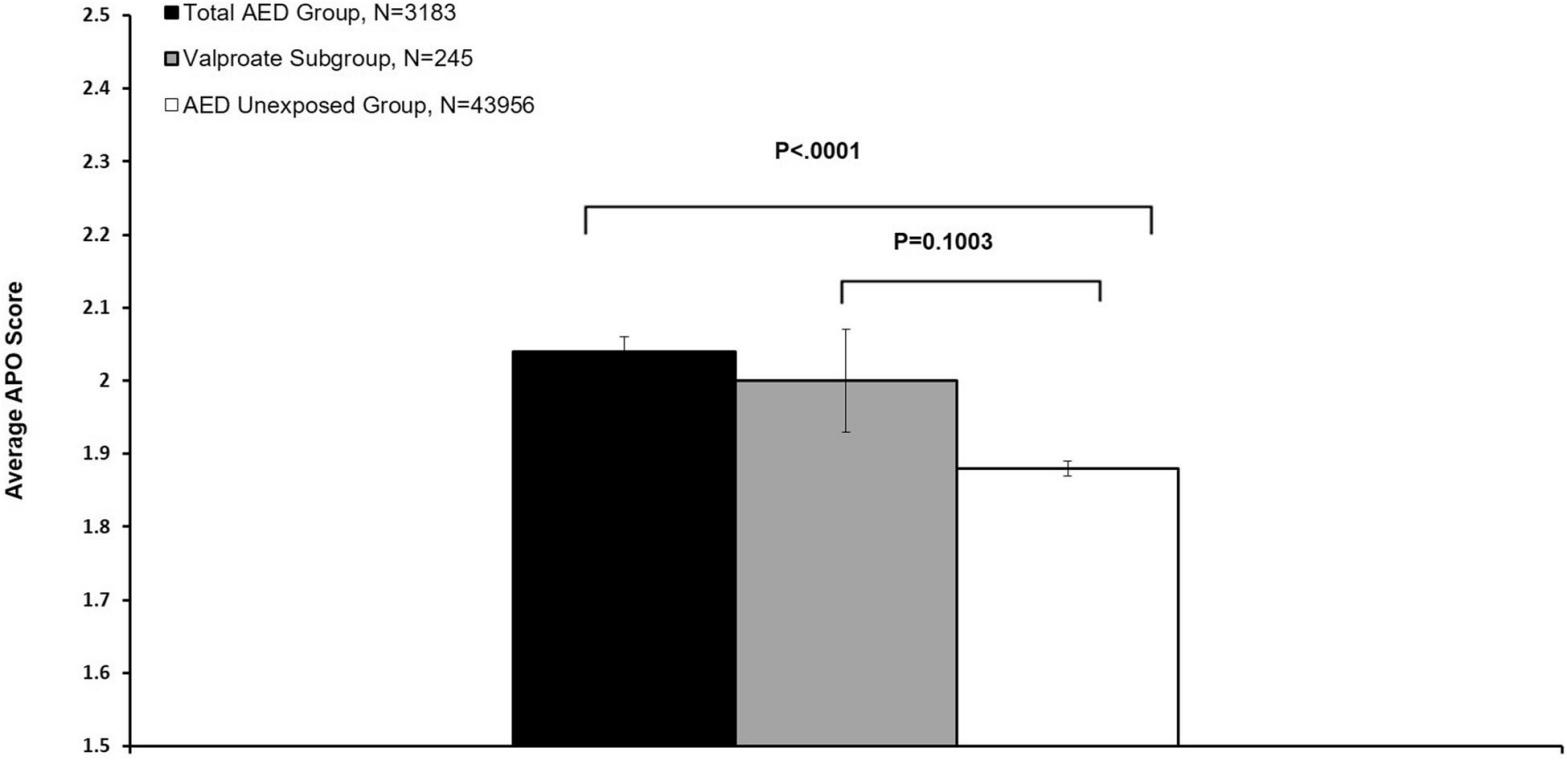
Comparison of Adverse Perinatal and Pregnancy Outcome (APO) Scores between Total AED Group, Valproate Subgroup, and AED unexposed group. Total AED group includes the patients using valproate

The combined outcome, APO scores were compared between AED, valproate only, and AED unexposed group (Fig. 1). The average APO score in the total AED group was significantly different for AED unexposed group (Mean ± SE: 2.04 ± 0.02 vs 1.88 ± 0.01, *P* < .0001), but not for the valproate subgroup (Mean ± SE: 2.00 ± 0.07 vs. 1.88 ± 0.01, *P* = 0.1003). The valproate subgroup (*n* = 226) was smaller than the total AED group (*n* = 3183), which could have affected the statistical results due to insufficient power.

**Fig. 2:**
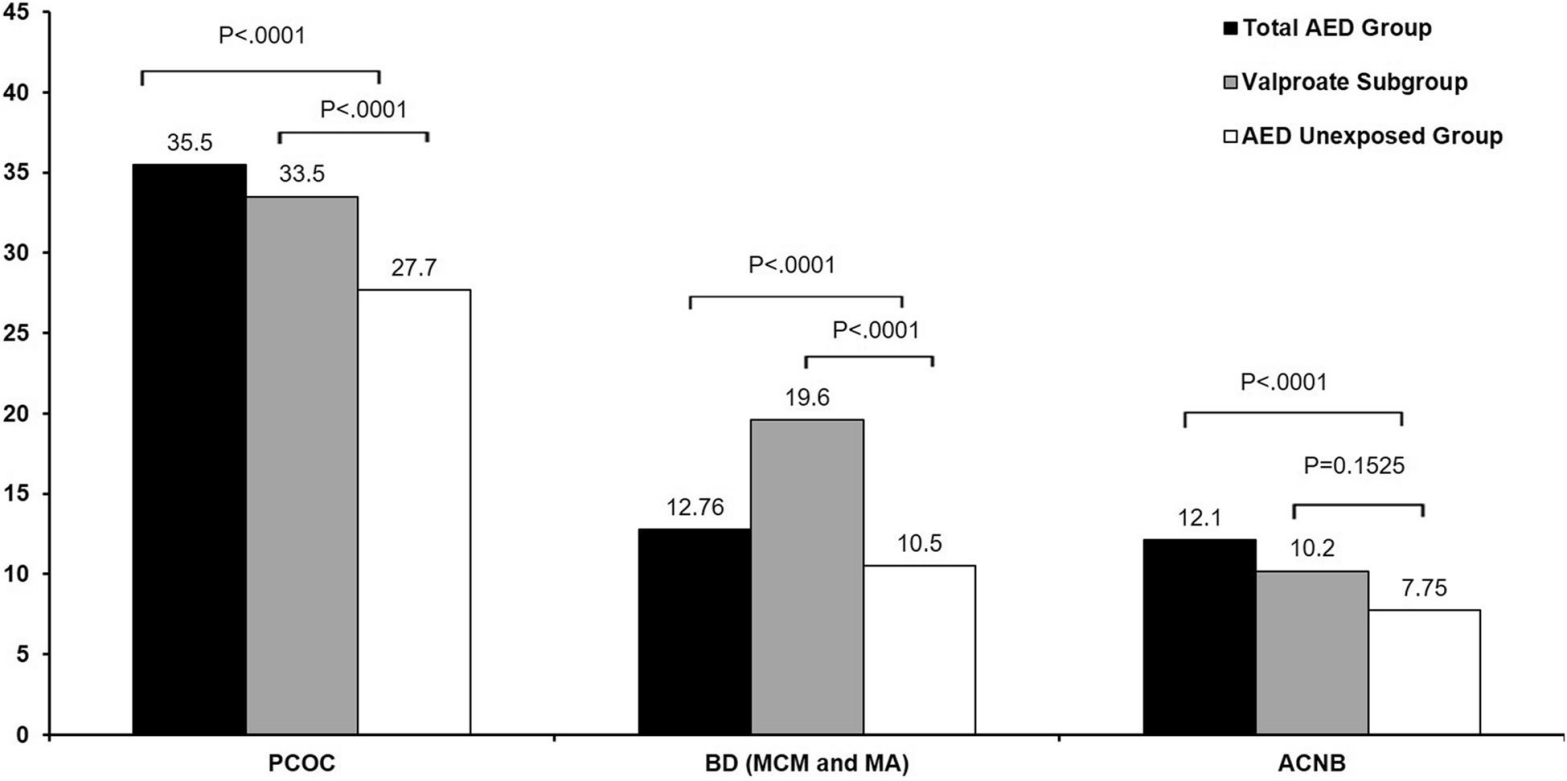
PCOC, Birth Defects (Major and Minor Congenital Malformation), and ACNB in the Total AED Group and Valproate Subgroup, and AED unexposed group. Total AED group includes patients using valproate. BD: Birth defects. ACNB: Abnormal condition of new born. PCOC: Pregnancy and obstetrical complication. LBW: Low birth weight

Figure 2 presents the incidence rates of PCOC, BD (MCM and MA), and ACNB in three study groups. Compared to AED unexposed group, the total AED exposed group had significant higher rates on PCOC (36% vs. 28%, *P* < .0001) and ACNB (12.1% vs. 7.8%, *P* < .0001). The rate of PCOC was not significantly higher in the valproate subgroup compared to the AED unexposed group (34% vs. 28%, *P* = 0.0509). The valproate subgroup had the highest rates of BD, significantly higher than the AED unexposed group (20% vs. 10.5%, *P* < .0001). ACNB in valproate subgroup was not different than the AED unexposed group (10.2% vs. 7.8%, *P* = 0.1525).

**Fig. 3:**
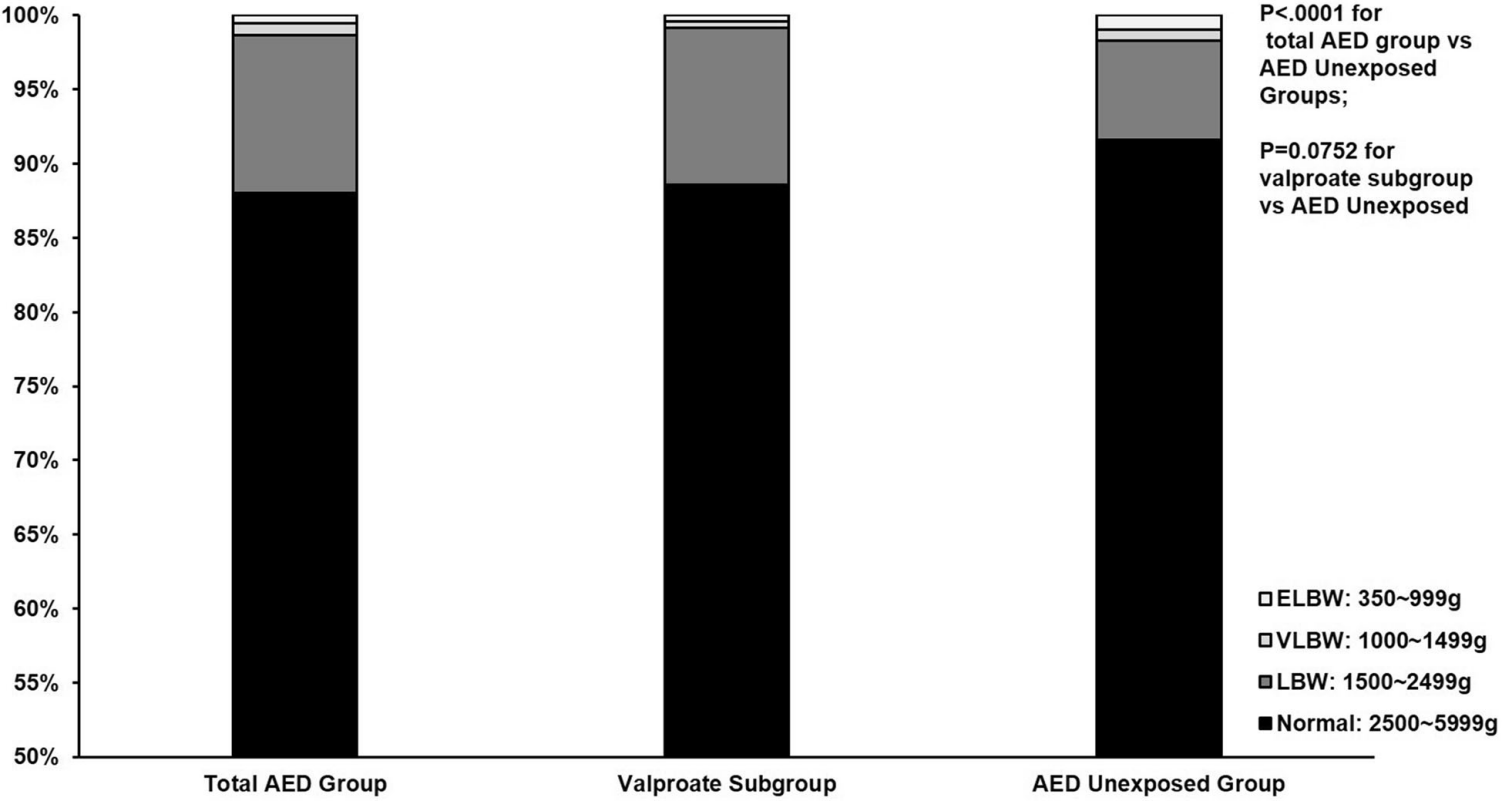
Distribution of Four Birth Weight Categories in the Total AED Group, Valproate Subgroup, and AED Unexposed Group. The total AED group includes patients using valproate. ELBW: Extreme Low Birth Weight. VLBW: Very Low Birth Weight. LBW: Low Birth Weight

Figure 3 delineates the distribution of four BW categories (Normal: 2500–5999 g, LBW: 1500 ~ 2500 g, VLBW: 1000 ~ 1500 g, and ELBW: <1000 g) in three study groups. The rate of LBW in the total AED exposed group was significantly higher than that of the AED unexposed group (88.1% vs. 91.6%, 10.6% vs. 6.7%, 0.9% vs. 0.7%, 0.5% vs. 0.99%, *P* < .0001). The valproate subgroup did not differ significantly in the distribution of BW categories from AED unexposed group (88.6% vs. 91.6%, 10.6% vs. 6.7%, 0.4% vs. 0.7%, 0.4% vs. 0.99%, *P* = 0.0752).

In Table 2, we further compared the propensity score adjusted drug effects and clinical importance (defined as Spearman correlation with the length of hospital stay during delivery) for each component or combined outcome in the total AED group or valproate subgroup. The adjusted drug effects and Spearman correlation with the length of hospital stay during delivery were significant for the total AED group on all four component outcomes, and for the valproate subgroup on BD and PCOC, but not for ACNB or LBW. The combined outcome APO was significantly associated with exposure to total AED (β ± SE: 0.24 ± 0.03, *P* < .0001) or valproate only (β ± SE: 0.32 ± 0.09, *P* = .0004).

**Table 2:** Propensity Score Adjusted Drug Effects, and Importance of Each Component or Combined Outcome in Pregnant Women Exposed to Total AED or Valproate Only

	Component Outcomes	Propensity Score Adjusted Drug Effects (β ± SE, *P* Value)	Spearman Correlation with the Length of Hospital Stay during Delivery (95%CI, *P* Value)
Total AED Group	BD	0.34 ± 0.16, *P* = 0.0356	0.08 (0.05 ~ 0.12, *P* < .0001)
	ACNB	0.60 ± 0.15, *P* = 0.0001	0.08 (0.04 ~ 0.11, *P* < .0001)
	PCOC	0.70 ± 0.11, *P* < .0001	0.24 (0.20 ~ 0.27, *P* < .0001)
	LBW	0.10 ± 0.02, *P* < .0001	0.13 (0.09 ~ 0.16, *P* < .0001)
	APO	0.24 ± 0.03, *P* < .0001	0.24 (0.20 ~ 0.27, *P* < .0001)
Valproate Subgroup	BD	0.96 ± 0.41, *P* = 0.0196	0.17 (0.05 ~ 0.29, *P* = 0.0066)
	ACNB	0.67 ± 0.43, *P* = 0.1223	0.02 (−0.10 ~ 0.15, *P* = 0.7135)
	PCOC	0.99 ± 0.32, *P* = 0.0019	0.16 (0.03 ~ 0.28, *P* = 0.0122)
	LBW	0.10 ± 0.06, *P* = 0.0856	0.04 (−0.09 ~ 0.16, *P* = 0.5640)
	APO	0.32 ± 0.09, *P* = 0.0004	0.16 (0.03 ~ 0.28, *P* = 0.0121)

Covariates include: mother’s epilepsy diagnosis, mother’s anxiety diagnosis, mother’s bipolar diagnosis, mother’s mental disorder diagnoses, mother’s mental disorder diagnoses, mother’s infection and parasitic diagnosis, mother's age, father's age, mother’s education level, father’s education level, mother’s total number of prenatal visits, mother’s parity, mother’s marital status, mother’s previous gestational diabetes, mother’s previous gestational hypertension, mother’s previous cesarean

*BD* Birth defects, *ACNB* Abnormal condition of new born, *PCOC* Pregnancy and obstetrical complication, *LBW* Low birth weight, *APO* Adverse Perinatal and Pregnancy Outcome

Expected and observed frequencies and percentages of each combination of the four observed outcomes were enumerated in Additional file 1: Table S3. Additional file 1: Table S3 also presents the estimated posterior mean *ŝ* and final estimate APO for 32 combinations of four component outcomes, each of which is associated with an unique score of APO, ranging from 1 to 8.

There was no correlation between the four component outcomes after controlling for the latent variable. Thus, local independence of the four component outcomes was established according to Yen’s Q_3_ Statistics. The internal homogeneity was confirmed in all four component outcomes. They all significantly correlate with each other and the combined outcome APO. APO was significantly correlated with the length of hospital stay during delivery (Rho = 0.27, *P* < .0001), and no correlation with infant breast fed status (Rho = −0.07, *P* < .0001) indicate that APO was associated with a well-established health status measure. The higher the APO score, the longer the hospital stay for the mothers and infants during delivery.

### Sensitivity study

We re-defined the pregnancy period calculating gestational age +10 day, 20 days, and 30 days to examine the change in association between AED exposure during pregnancy and four component outcomes. There were no significant differences between these time windows.

## Discussion

The total AED group was significantly different from the AED unexposed group on all observed outcomes, whereas valproate subgroup differed statistically from AED unexposed group only on BD and PCOC. These two exposure groups had varied patterns of observed outcomes that were combined using the latent trait model. The psychometric properties of the combined outcome were evaluated and compared among the two exposed groups and one healthy comparison group. The four component outcomes were found to be not significantly different on the incidence rates. One exception was PCOC, which had the highest incidence out of the three component outcomes. However, neither the differences of AED effects on PCOC nor the correlation between PCOC and the length of the hospital stay during delivery was significantly different between the total AED group and AED unexposed group. Thus, these four component outcomes exhibited a high level of homogeneity and demonstrated the validity of component selection for the AED safety study.

Table 1 provides evidence for a pronounced difference between the mother-infant pairs exposed to AEDs versus AED unexposed group and raises a concern that studies of pregnancy outcome need to control for these differences. In our study, propensity score was used to adjust these covariates for drug effect assessment.

Figures 1, 2, and 3 raise concern for combining outcomes in valproate drug safety studies. Compared to the AED unexposed group, AED use in the total exposed group was associated with significant effects on all four component outcomes, whereas, valproate use was related to increased BD and PCOC, and had no significant effect on ACNB and BW. The lack of differences for valproate on APO, ACNB and BW may be in part due to the small sample size of the valproate subgroup. Previous studies using birth registry data has shown that fetal valproate exposure is associated with higher rates of BD than other AEDs. For example, the UK Birth Registry reported a 6.7% rate of major congenital malformations for valproate, and the North American AED Pregnancy Registry reported a rate of 10.7% [3, 45]. However, the incidence of minor abnormalities in our study, 9.1% in AED unexposed group and 10.7% in the total AED group, was lower than reported in the literature, 15% to 20% in the general population and 37% in AED exposed pregnant women [46–50]. Considering the difficulties of identifying minor abnormalities, under-reporting or misdiagnosis of minor abnormalities in claims data might account for this discrepancy. Given that valproate exposure is not consistently associated with the four component outcomes and violates the criteria for component selection for a composite outcome, a concern is raised about the validity of combining these four outcomes in a valproate safety study.

To our knowledge, combining outcomes using a latent variable model has not been utilized in any pharmaco-epidemiological studies previously. This model was first described in 2008 for combining four birth defect outcomes to construct an infant morbidity index [11]. We employed the model to assess the comprehensive effects of AEDs on four adverse perinatal and pregnancy outcomes in both mothers and infants. Superior to other composite outcomes, the latent variable model generates a continuous measure that correlates to the component outcomes with different levels and takes into account the comprehensive effects of all component outcomes [11].

The final estimate of the latent variable *S* ^^^ ranged from 0.08 for normal infant-mother pairs to 0.61 for the mother-infant pairs with different patterns of BD, ACNB, PCOC, and ELBW. These estimates are similar in magnitude to those documented previously [11].

This article is based on a thesis published by one of the authors in 2013 (http://ufdc.ufl.edu/UFE0046207/00001).

### Study limitations

Several limitations should be considered as a consequence of using linked claims data and the nature of the study. First, by combining MCM, MA, LBW, and PCOC, the latent variable APO is an overall adverse outcome for both mothers and infants. The association between drug exposure and each individual component outcome is unknown if latent variable APO is used as a dependent variable in the model. Second, the power to detect differences in the valproate only subgroup is a concern due to small sample size. The insignificant difference in APO between valproate only subgroup and health unexposed group might be due to the inadequate statistical power. Third, MAs might be underestimated in this study, which could cause underestimation of APO score. However, the misclassification of MAs is non-differential, so it should not affect the assessment of differences between drug use groups. Finally, this latent variable model combines manifest outcomes based upon the probability of occurrence in the study population. The severity of each outcome is not mathematical weighted in the combining process. Future studies are needed to develop more advanced statistical models to combine more specific outcomes based upon not only the probability of occurrence, but also the severity of each outcome.

## Conclusions

This study used a latent trait model to assess adverse pregnancy and perinatal outcomes in women exposed to antiepileptic drugs during pregnancy. We recommend using this latent trait model in other drug studies examining similarly related component outcomes. If the study drug, is only weakly associated with any of the selected component outcomes, the study drug’s effects on the combined outcome may be diluted and be statistically non-significant compared to the reference group. Such an approach is detrimental to any drug safety study as the results move towards the null and the true teratogenic effects of the drug can be masked. Hence, evaluation of selected components is essential before a latent trait model can be used to assess a combined outcome.

## Additional file

Additional file 1: Table S1. Operational Definition 625 for Component Outcomes. **Table S2.** Demographic Characteristics and Missing Data of Study Participants. **Table S3.** Observed Frequency (OBFREQ), Expected Frequency (EXFREQ), Observed Percents (OB%), Expected Percents (EX%), Estimates of Posterior Mean of the Latent Variable *S* ̂ 632 and the APO by Combinations of Four Observed Outcomes. (DOCX 23 kb)

## Abbreviations

ACNB: Abnormal Condition of Newborn
AED: Antiepileptic Drugs (also called Anticonvulsant Drugs)
APO: Adverse Perinatal Outcome
BD: Birth defect
BW: Birth weight
CI: Confidence Interval
ELBW: Extremely Low Birth Weight
LBW: Low birth weight
MA: Minor anomaly
MCM: Major congenital malformation
NBW: Normal Birth Weight
PCOC: Pregnancy and Obstetric Complication
SE: Standard Error
VLBW: Very Low Birth Weight
