# Supplementary material for: Combining adverse pregnancy and perinatal outcomes for women exposed to antiepileptic drugs during pregnancy, using a latent trait model
Source: BMC Pregnancy Childbirth. 2017 Jan 6;17:10. doi: 10.1186/s12884-016-1190-7 (PMC5219655; doi:10.1186/s12884-016-1190-7)
Supplement: Additional file 1: Table S1. — Operational Definition 625 for Component Outcomes. Table S2. Demographic Characteristics and Missing Data of Study Participants. Table S3. Observed Frequency (OBFREQ), Expected Frequency (EXFREQ), Observed Percents (OB%), Expected Percents (EX%), Estimates of Posterior Mean of the Latent Variable S ̂ 632 and the APO by Combinations of Four Observed Outcomes. (DOCX 23 kb) [file 12884_2016_1190_MOESM1_ESM.docx]

**Additional file 1**

Table S1. Operational Definition for Component Outcomes

| Variable | Operational Definition |
| --- | --- |
|  |  |
| Birth Defects, including Congenital Major Malformations and Minor Anomalies | at least one of the ICD-9 codes: 740.xx to 759.xx, 710.xx to 739.xx, 520.xx to 629.xx, 320.xx to 389.xx, 140.xx to 239.xx, or 279.xx on hospital inpatient or outpatient discharge claims any time from infant birth date to 365 days of follow up. |
| Abnormal Condition of New Born | Checked ‘Yes‘ on any of the following checkboxes on birth certificate:  AC_VENT-IMMED  AC_VENT_30MIN  AC_VENT_MORE_6HOUR  AC_NICU  AC_SURFACTANT  AC_ANTIBIOTIC_SEPSIS  AC_SEIZURE  AC_OTHER  AC_HYALINE_MEM  AC_BIRTH_INJURY |
|  | Or at least one of the ICD-9 codes 280.x-289.x, 767.x, 760.7x, 779.5, 769.x, 770.1x, 779.0, 345.x on hospital inpatient or outpatient discharge claims any time from infant birth date to 365 days of follow up. |
| Birth Weight | Used BIRTH_WEIGHT_GRAMS from BVS data and categorized to 4 levels:  extremely Low BW (≥350 and <999 g), very Low BW (≥1000 and <1499 g), Low BW (≥1500 and <2499 g),  normal BW (≥2500 and <5999 g). |
| Pregnancy Complications  Placenta previa, w/o bleeding, unspec.  Placenta previa, v/bleeding, unspec.  Abruptio placetae, unspec  Hemorrhage in pregnancy, unspec.  Gestational hypertension  Mild or unspecified pre-eclampsia  Severe pre-eclampsia  Eclampsia, unspec.  Obstetrical Complications  Cesarean delivery  Forceps or vacuum extractor delivery  Postpartum hemorrhage  Preterm Birth | Identified at least one of the following ICD-9 or CPT codes:  641.0  641.1  641.2  641.9  642.3, 642.9  642.4  642.5  642.6  Checked ‘Yes‘ on any of the following checkboxes from BVS data and at least one of the ICD-9 or CPT codes:  MD_CES_LABOR_ATTEMPT or 669.7, 763.4, 59510, 59514, 59515, 59612  MD_VAGINAL_FORCEPS, MD_VAGINAL_VACUUM, or 763.2, 763.3, 669.51  666.xx  Gestational age <= 37 weeks |

Table S2. Demographic Characteristics and Missing Data of Study Participants.

| Characteristics | All Mother-Infant Pairs Included in This Study  N=47,139 |
| --- | --- |
| Maternal age at infant born, Mean ± SD | 24.8±5.3 |
| Father’s age at infant birth, Mean ± SD | 43.6±29.8 |
| Mother’s Race, N(%)  White  Black  Others  Missing | 22533(48)  14420 (31)  10102 (21)  84 (0.2) |
| Father’s Race, N(%)  White  Black  Others  Missing | 17124 (36)  10060 (21)  8498 (18)  11457 (24) |
| Father’s education level, N(%)  Above High School  Missing | 14,984 (32)  10,111 (21) |
| Mother’s previous adverse pregnancy experience, N(%)  Missing, N(%) | 330 (0.7)  27336 (58) |
| Mother’s prenatal use, N(%)  Missing, N(%) | 20762 (44)  26090 (55) |
| Mother’s total number of prenatal visits, Mean ± SD  Missing, N(%) | 8.9 ± 15.8  2024 (4) |
| Mother’s marital status, Yes, N(%)  Missing, N(%) | 19,178 (41)  2 (0) |
| Mother’s parity, Mean ± SD | 1.7 ± 3.8 |
| Mother’s tobacco use, N(%)  Missing, N(%) | 8069 (17)  538 (1) |
| Mother’s average tobacco use, Mean ± SD  Missing, N(%) | 1.8 ± 9.3  928 (2) |
| Mother’s alcohol use, N(%)  Missing, N(%) | 213 (0.5)  37 (0.1) |
| Mother’s education level, N(%)  Above High School  Missing | 17,945 (38)  868 (2) |
| Infant male gender, N(%)  Missing, N(%) | 20,297 (44)  6910 (15) |
| Infant breast fed, N(%)  Missing, N(%) | 13,034 (28)  27,441 (58) |
| Mother’s previous gestational diabetes, N(%)  Missing, N(%) | 688 (3.5)  27,336 (58) |

| Characteristics | All Mother-Infant Pairs Included in This Study  N=47,139 |
| --- | --- |
| Mother’s previous gestational hypertension, N(%)  Missing, N(%) | 1378 (3)  3335 (7) |
| Mother’s previous eclampsia, N(%)  Missing, N(%) | 127 (0.3)  3335 (7) |
| Mother’s previous preterm, N(%)  Missing, N(%) | 992 (2.3)  3335 (7) |
| Mother’s previous other risk factors, N(%)  Missing, N(%) | 7668 (16)  3335 (7) |
| Mother’s previous cesarean, N(%)  Missing, N(%) | 3362 (7)  27336 (58) |

Table S3. Observed Frequency (OBFREQ), Expected Frequency (EXFREQ), Observed Percents (OB%), Expected Percents (EX%), Estimates of Posterior Mean of the Latent

Variable $\hat{S}$ and the APO by Combinations of Four Observed Outcomes.

| BD | ACNB | PCOC | BW | OB-FREQ | EX-FREQ | OB% | EX% | $\hat{S}$ | APO |
| --- | --- | --- | --- | --- | --- | --- | --- | --- | --- |
| Yes | Yes | Yes | 350–999 | 105.00 | 47.94 | 0.22 | 0.10 | 0.61 | 7.98 |
| Yes | Yes | Yes | 1000–1499 | 85.00 | 34.60 | 0.18 | 0.07 | 0.44 | 5.50 |
| Yes | Yes | Yes | 1500–2499 | 196.00 | 136.31 | 0.42 | 0.29 | 0.31 | 3.87 |
| Yes | Yes | Yes | 2500–5999 | 201.00 | 135.68 | 0.43 | 0.29 | 0.19 | 2.39 |
| Yes | Yes | No | 350–999 | 1.00 | 1.44 | 0.00 | 0.00 | 0.56 | 6.95 |
| Yes | Yes | No | 1000–1499 | 2.00 | 2.37 | 0.00 | 0.01 | 0.44 | 5.45 |
| Yes | Yes | No | 1500–2499 | 22.00 | 29.76 | 0.05 | 0.06 | 0.28 | 3.52 |
| Yes | Yes | No | 2500–5999 | 301.00 | 160.85 | 0.64 | 0.34 | 0.14 | 1.76 |
| Yes | No | Yes | 350–999 | 80.00 | 38.75 | 0.17 | 0.08 | 0.57 | 7.13 |
| Yes | No | Yes | 1000–1499 | 60.00 | 48.79 | 0.13 | 0.10 | 0.44 | 5.47 |
| Yes | No | Yes | 1500–2499 | 274.00 | 394.20 | 0.58 | 0.84 | 0.29 | 3.65 |
| Yes | No | Yes | 2500–5999 | 1006.00 | 1084.62 | 2.13 | 2.30 | 0.16 | 2.01 |
| Yes | No | No | 350–999 | 1.00 | 1.34 | 0.00 | 0.00 | 0.54 | 6.71 |
| Yes | No | No | 1000–1499 | 1.00 | 3.41 | 0.00 | 0.01 | 0.43 | 5.41 |
| Yes | No | No | 1500–2499 | 77.00 | 103.66 | 0.16 | 0.22 | 0.26 | 3.29 |
| Yes | No | No | 2500–5999 | 2608.00 | 2604.98 | 5.53 | 5.53 | 0.10 | 1.28 |
| No | Yes | Yes | 350–999 | 130.00 | 105.35 | 0.28 | 0.22 | 0.59 | 7.36 |
| No | Yes | Yes | 1000–1499 | 102.00 | 99.59 | 0.22 | 0.21 | 0.44 | 5.49 |
| No | Yes | Yes | 1500–2499 | 576.00 | 539.30 | 1.22 | 1.14 | 0.30 | 3.77 |
| No | Yes | Yes | 2500–5999 | 697.00 | 834.06 | 1.48 | 1.77 | 0.18 | 2.22 |
| No | Yes | No | 350–999 | 0.50 | 3.41 | 0.00 | 0.01 | 0.55 | 6.82 |
| No | Yes | No | 1000–1499 | 3.00 | 6.88 | 0.01 | 0.01 | 0.43 | 5.43 |
| No | Yes | No | 1500–2499 | 77.00 | 127.93 | 0.16 | 0.27 | 0.27 | 3.42 |
| No | Yes | No | 2500–5999 | 1295.00 | 1320.85 | 2.75 | 2.80 | 0.12 | 1.55 |
| No | No | Yes | 350–999 | 134.00 | 89.72 | 0.28 | 0.19 | 0.56 | 6.97 |
| No | No | Yes | 1000–1499 | 83.00 | 141.25 | 0.18 | 0.30 | 0.44 | 5.45 |
| No | No | Yes | 1500–2499 | 1477.00 | 1642.46 | 3.13 | 3.48 | 0.28 | 3.55 |
| No | No | Yes | 2500–5999 | 8117.00 | 7863.94 | 17.22 | 16.68 | 0.14 | 1.81 |
| No | No | No | 350–999 | 1.00 | 3.27 | 0.00 | 0.01 | 0.53 | 6.62 |
| No | No | No | 1000–1499 | 1.00 | 9.95 | 0.00 | 0.02 | 0.43 | 5.40 |
| No | No | No | 1500–2499 | 586.00 | 473.60 | 1.24 | 1.00 | 0.25 | 3.18 |
| No | No | No | 2500–5999 | 28841.0 | 29049.74 | 61.18 | 61.62 | 0.08 | 1.06 |

Note: BD: Birth defects.

ACNB: Abnormal condition of new born.

PCOC: Pregnancy and obstetrical complication.

LBW: Low birth weight.

APO: Adverse Perinatal and Pregnancy Outcome
